# Time to consider sequencing anti-inflammatory treatments with chemotherapy and immuno-stimulation?

**DOI:** 10.3389/fimmu.2022.1082142

**Published:** 2022-12-22

**Authors:** George Blanck

**Affiliations:** ^1^ Department of Molecular Medicine, Morsani College of Medicine, University of South Florida, Tampa, FL, United States; ^2^ Department of Immunology, H. Lee Moffitt Cancer Center and Research Institute, Tampa, FL, United States

**Keywords:** immunoediting, CDR3-antigen chemical complementarity scoring, granzyme B, immune checkpoint blockade, cancer and inflammation

## Introduction

Immuno-stimulatory therapies, such as immune checkpoint blockade (ICB), often presuppose the availability of a T-cell receptor (TCR) that can bind a cancer antigen or available antibodies to complete an antibody-dependent cell-mediated cytotoxicity process. However, with selection against cancer cells expressing the antigen, and with limitations on TCR or antibody or antigen diversity, immuno-stimulation, i.e, immuno-stimulation that requires an adaptive immune component, should eventually fail. In addition, it is clear that certain inflammatory settings can support cancer growth, e.g., Crohn’s disease. Thus, how to know when to begin an anti-inflammatory arm of a treatment plan but to not interfere with an active anti-cancer adaptive immune response?

## Developing parameters for application of anti-inflammatory treatments

Paderi et al. (1) have shown that corticosteroid treatments soon after an ICB treatment, for several solid tumor types, have no impact on survival, but the same treatments delayed are associated with better progression-free survival. Possibly, after a certain period, TCR-cancer antigen interactions have subsided, and the remaining immune microenvironment only serves to facilitate tumor growth.

Our group has suggested a second, potential indicator for corticosteroid use in the low grade glioma (LGG) setting, by identifying patients that are not likely to be benefitting from productive adaptive immune receptor (IR)-antigen interactions (2, 3). In one LGG dataset, patients that lack a demonstrable, productive, chemically complementary, IR-candidate antigen interaction, and who have been treated with corticosteroids, have a greater lifespan than those patients with the same IR-antigen, chemical complementarity deficits but no corticosteroid treatments (4). Thus, a key hope would be a prospective clinical trial whereby anti-inflammatory treatments would occur at selected time periods following ICB or other immuno-stimulatory treatments, with TCR-mediated activation assessed, during the course of both the ICB and anti-inflammatory treatment periods.

Considering a likely more immediate and practical possibility, it is also important to note that in several settings, low granzyme B levels have been correlated with ICB failure (5–8), which raises the question of whether microscopic staining for granzyme B before or following ICB treatment should be also be considered as a parameter for an anti-inflammatory treatment course? Low granzyme B levels likely represent a lack of T-cell activation, in turn due to a lack of TCR binding to an epitope. In such cases, the immune microenvironment referred to above, supporting tumor growth (1, 4), could be in effect.

It should be noted that all three of the above parameters (time period, TCR-antigen complementarity scoring, granzyme B levels) would presumably be only indirectly reflective of specific, pro-tumor cell proliferation mediators. However, while several of these immune-related mediators are known, such as Interleukin-8 (CXCL8) (9, 10), and have been considered as clinical targets for decades, no clinical approaches to mitigating the impact of such mediators have been adopted. Thus in the meantime, the indirect correlates of a failed adaptive immune response, with a presumptive, pro-tumor immune microenvironment left behind, may be the best bet for timing the application of broad based anti-inflammatory drugs, which as noted above, do correlate with positive outcomes in some cases. Furthermore, there has been extensive experience with the use of anti-inflammatory treatments to reduce adverse effects of immuno-stimulation, and thus, considering the inspiration of Paderi et al. (1), there may already be extensive records that could be mined for further guidance on anti-inflammatory treatment timing.

## In sum

A likely long-term hope would be data-driven bases to repeatedly apply the following regimens for maintenance: (i) neoantigen-generating chemo, (ii) immuno-stimulation, and (iii) anti-inflammation.

## Author contributions

The author confirms being the sole contributor of this work and has approved it for publication.
